# Biosynthesis and characterization of silver nanoparticles from *Punica granatum* (pomegranate) peel waste and its application to inhibit foodborne pathogens

**DOI:** 10.1038/s41598-023-46355-x

**Published:** 2023-11-09

**Authors:** Salma M. Farouk, Samah H. Abu-Hussien, Basma T. Abd-Elhalim, Reham M. Mohamed, Naira M. Arabe, Ahmed A. T. Hussain, Mostafa E. Mostafa, Bahaa Hemdan, Salwa M. El-Sayed, Ashraf Bakry, Naglaa M. Ebeed, Mahmoud Salah, Hesham Elhariry, Ahmed Galal

**Affiliations:** 1 Undergraduate student, Biotechnology Program, Faculty of Agriculture, Ain Shams University, Hadayek Shoubra, PO Box 68, Cairo, 11241 Egypt; 2 Department of Agricultural Microbiology, Faculty of Agriculture, Ain Shams University, Hadayek Shoubra, PO Box 68, Cairo, 11241 Egypt; 3 Environmental and Climate Change Research Institute, National Research Center, Giza, 1266 Egypt; 4 Department of Biochemistry, Faculty of Agriculture, Ain Shams University, Hadayek Shoubra, PO Box 68, Cairo, 11241 Egypt; 5 Department of Genetics, Faculty of Agriculture, Ain Shams University, Hadayek Shoubra, PO Box 68, Cairo, 11241 Egypt; 6 Department of Environmental Agricultural Science, Faculty of Graduate Studies and Environmental Research, Ain Shams University, Cairo, 11566 Egypt; 7 Prevention and Detection of Microbial and Chemicals Contamination in Food Lab, School of Food and Biological Engineering, Jiangsu University, Zhenjiang, 212013 Jiangsu China; 8 Department of Food Science, Faculty of Agriculture, Ain Shams University, Hadayek Shoubra, PO Box 68, Cairo, 11241 Egypt; 9 Department of Poultry Production, Faculty of Agriculture, Ain Shams University, Hadayek Shoubra, PO Box 68, Cairo, 11241 Egypt

**Keywords:** Biochemistry, Biotechnology, Microbiology

## Abstract

Polyphenolics have been predicted to effectively develop antimicrobial agents for the food industry as food additives and promote human health. This study aims to synthesize pomegranate peel extract (PPE) with silver nanoparticles (AgNPs) against eight foodborne pathogens. Multispectroscopic analysis of UV–vis spectroscopy, Zeta potential, Fourier transform infrared (FTIR) and scanning electron microscopy (SEM) analysis were used to characterize the interaction between PPE and AgNPs. Eight foodborne pathogenic strains (six bacterial and two fungal strains) *Bacillus subtilis* ATCC 6633, *Enterococcus faecalis* ATCC 29212, *Escherichia coli* ATCC 8379, *Klebsiella pneumoniae* ATCC 00607, *Salmonella typhi* DSM 17058, *Shigella sonnei* DSM 5570, *Aspergillus flavus* ATCC 9643, and *Rhizopus oryzae* ATCC 96382 were used to test the inhibitory potential of PPW-AgNPs. The reaction colour of PPE-AgNPs from yellow to brown indicated that the nanoparticles were successfully formed. The UV absorption of PPE-AgNPs was detected at 440 nm of 0.9 SPR. SEM image of PPE-AgNPs exhibited spherical shapes with a zeta potential of − 20.1 mV. PPE-AgNPs showed high antimicrobial activity against all tested strains. The highest inhibition activity of PPE-AgNPs was recorded for the *B. subtilis* strain followed by *K. pneumonia*, while the highest resistance was noticed for *R. oryzae.* The components of pomegranate peel were analyzed using gas chromatography–mass spectrometry (GC–MS). The major constituents of pomegranate peel is phenol (51.1%), followed by Isocitronellol (19.41%) and 1-Propanol, 2-(2-hydroxypropyl)- (16.05%). PPE is key in the simple, eco-friendly green synthesis of extracellular stable AgNPs as an alternative source for harmful chemical disinfectants.

## Introduction

Food pathogenic bacteria or fungi are considered a common international health problem that causes many severe diseases and deaths worldwide. Polyphenolics have been predicted to be effective in developing antibacterial agents for the food industry as food additives and for human use. Polyphenolics inhibit the spore germination of pathogens (bacteria and fungi) in food and plants. The pomegranate (*Punica granatum* L.) is an edible fruit cultivated extensively in many tropical and subtropical nations^1^. This fruit is considered beneficial to the human diet. In the pomegranate juice industry, about 50% of the weight of a pomegranate is made up of its peel, a significant source of polyphenolic compounds^2^. Pomegranate peels were used to treat many human diseases, such as diarrhoea, vomiting, headaches, and other disorders, such as colitis, dysentery, and ulcers^3^. Pomegranate peel extract (PPE) has many biological activities, such as antioxidant, anti-inflammatory, and anti-carcinogenic properties, used to treat cancer, dental infections, bacterial wounds, etc*.* The bioactive chemicals found in pomegranate peel extract (PPE) are credited with having both antioxidant and antibacterial properties^4^. PPE is reported to contain numerous phenolic compounds, such as ellagic acid and its compounds, ellagitannins like punicalin and punicalagin, flavonoids, alkaloids, and phenolic acid, which possess antimicrobial properties^5,6^. A study also showed that PPE provided significant amounts of bioactive chemicals, thus considered a superior antibacterial agent^7^. Developing PPE based on nanocomposites with antibacterial materials such as nanoparticles, natural polymers, and biomolecules is an important research area.

In this context, nanoparticles (NPs) have exclusive physical and chemical characteristics superior to their inhibitory potential against pathogens. AgNPs can inhibit microbial growth and foodborne pathogens such as *Staphylococcus aureus, Escherichia coli, Aspergillus niger* and *Rhizobus nigricans*^8^*.* AgNPs have a high surface area/volume ratio, which increases as the particle size decreases^9^. It is well known that AgNPs have the smallest size and the highest inhibitory activity against tested pathogens, but it is still unclear^10^. A previous study reported that triangular particles of AgNPs have higher inhibitory activity than elongated and spherical particles^11^. The antimicrobial ability of AgNP is affected by several factors such as oxygen existence, pH and temperature^12^. For instance, the acidity could enhance AgNP activity by binding to sulfur or phosphorus groups (-P, -S or -SH)^13^. In this context, the AgNP mode of action is attributed to binding with phosphorus and sulfur in the DNA structure inside the cells. Upon interaction with DNA, AgNP caused cellular leakage of metabolites, destabilization and cell death^13,14^. Moreover, binding silver particles to the enzymes inside the cells increases reactive oxygen that denatures the cells' protein and uncontrols the transportation of ions, leading to cell death^15^. The biosynthesis method of AgNPs is considered an easy, quick, safe, predictable, and environmentally friendly way to fabricate well-defined sizes and morphology under optimal conditions for research^15^. The antimicrobial resistance (AMR) crisis has been attributed to the misuse of these drugs and the shortage of discovering new efficient and safe bio alternatives^16^. Therefore, the researchers resorted to producing highly efficient and environmentally friendly bio-active materials based on their antioxidant and antimicrobial properties instead of synthetic chemicals^17^. This study aims to evaluate pomegranate peel extract (PPE) loaded on silver nanoparticles (AgNPs) against eight foodborne pathogens. Multi-spectroscopic analysis of PPE-AgNPs driven via uv-spectroscopy, Fourier transform infrared (FTIR), particle size (nm), zeta potential (mv), poly-diversity index (PdI), and scanning electron microscope (SEM). The Inhibition activity of PPE-AgNPs was evaluated using minimum inhibitory concentration (MIC) and minimum lethal concentration (MLC) of food pathogenic strains (six bacteria and two fungal strains) including *Bacillus subtilis* ATCC 6633, *Enterococcus faecalis* ATCC 29212, *Escherichia coli* ATCC 8379, *Klebsiella pneumoniae* ATCC 00607, *Salmonella typhi* DSM 17058, *Shigella sonnei* DSM 5570, *Aspergillus flavus* ATCC 9643, and *Rhizopus oryzae* ATCC 96382.

## Materials and methods

### Materials

Silver nitrate (AgNO_3_) was purchased from Sigma Aldrich. Standard antibiotics (gentamicin 10.0 μg and cefuroxime 30.0 μg) were purchased from Novartis company in Egypt. Mueller–Hinton agar (MHA) media were obtained from an Elgomhoria supplier in Cairo, Egypt. Fresh pomegranate fruits were collected from local markets in Cairo, Egypt, according to the IUCN policy statement for collecting plant materials (https://portals.iucn.org/library/efiles/documents/PP-003-En.pdf). All chemicals were analytical grade. For used microorganisms, the antimicrobial activity of nanoparticles were tested against eight pathogenic microorganism strains including *Bacillus subtilis* ATCC 6633*, Enterococcus faecalis* ATCC 29212, *Escherichia coli* ATCC 8379, *Klebsiella pneumoniae* ATCC 00607, *Salmonella typhi* DSM 17058, *Shigella sonnei* DSM 5570, *Aspergillus flavus* ATCC 9643, and *Rhizopus oryzae* ATCC 96382. These strains were obtained from the Microbiology Department, Faculty of Agriculture, Ain Shams University. All obtained strains were maintained on glucose agar (Difco Manual, 1984) and kept in the refrigerator at 4 °C.

### Standard inoculum

Standard inoculum for all tested pathogenic bacterial and fungal strains was prepared according to a previous method^18^. A loop of freshly prepared culture of bacterial inoculum was inoculated with 50 mL glucose broth and incubated at 37 °C for 24 h using the shake flask method at 150 rpm. The spore suspension was inoculated with a broth of malt media and incubated at 28 °C for 72 h at 150 rpm.

### Preparation of pomegranate peel extract (PPE)

The pericarp was manually separated from pomegranate peels and used for extraction. According to a previous study^19^, the pomegranate peel aqueous extract was obtained. Twenty grams of fresh pericarp was soaked in 60.0 mL of distilled water and placed in the shaker at 150 rpm for 4 h. Samples were left in the dark at room temperature for 12 h and centrifuged at 10,000 rpm for 15 min. Supernatants were collected and used for subsequent studies.

### Biosynthesis of pomegranate peel extract (PPE) loaded on silver nanoparticles (AgNps)

Fifty millilitres of AgNO_3_ (1 mM) were dropwised into a glass beaker containing 2 mL of the previously prepared PPE solution (33.3% w/v in distilled water). Then, the reaction mixture was incubated overnight in the dark using a rotary shaker incubator at 200 rpm, and the temperature was set at 30 °C (Shin Saeng, South Korea). A negative control of pomegranate extract without a silver nitrate solution was prepared. The change in colour from pale yellow to dark orange indicated the formation of the PPE-AgNPs^20^.

### Characterization of PPE-AgNPs

Appropriate characterisation techniques analysed the functional properties of the fabricated nanoparticles. The functional groups binding between AgNPs with PPE were analysed by UV–vis spectrophotometer (UV Analyst-CT 8200) in the wavelength range from 200 to 800 nm, Fourier transform infrared spectroscopy (FTIR, Shimadzu, Tracer-100) in the range of 500–4000/cm, scanning electron microscopy (FE-SEM; JOEL JSM-7800F) operated at 15 kV. The prepared PPE-AgNPs were measured using a Zeta Analyzer (NICOMPTM 380 ZLS)^21^. All characterization investigations were performed using standard methods by Creative Egyptian Biotechnologists (CEB) company, https://www.ceb-eg.com/, Dokki, Giza, Egypt. UV–VIS and FTIR absorption spectra of PPE and PPE-AgNPs samples were plotted using OriginPro 2022 (64-bit) SR1 v9.9.0.2 software https://www.originlab.com/index.aspx?go=Support&pid=4440.

### Inhibitory activity of PPE-AgNPs

The antimicrobial activity of the biosynthesized PPE-AgNPs was tested against eight pathogenic microbial strains (6 bacteria and 2 fungi). *B. subtilis* ATCC 6633, *E. faecalis* ATCC 29212, *E. coli* ATCC 8379, *K. pneumoniae* ATCC 00607, *S. typhi* DSM 17058, *S. sonnei* DSM 5570, *A. flavus* ATCC 9643, and *R. oryzae* ATCC 96382 were grown on MHA and malt agar media, respectively. Well diffusion method was used to test the inhibitory effect^22^ of eight different concentrations of PPE-AgNPs against the eight microbial strains. Briefly, 50.0 μL of each microbial inoculum (10^6^ CFU/mL) was spread separately onto the surface of MHA agar plates. Wells were made using a sterilized cork porer 6 mm in diameter then filled with 0.1 mL of PPE-AgNPs in different concentrations (1000, 500, and 250, 125, 75, and 25 μg/mL) using ampicillin and streptomycin 1000 μg/mL a as positive control. All plates were incubated at 37 °C and 28 °C for 24 h and 72 h for bacteria and fungi, respectively. The zone of inhibition around each well was measured using a ruler and expressed by centimetre as IZD (cm)^23^.

Activity index was calculated according to^24^ which was obtained by comparing the diameter of the inhibition zone of PPE-AgNPs with the standard reference antibiotic according to the following equation:

$${Activity\; index \left( {AI} \right) = IZD \;of\; AgNPs}/{IZD \;of\; reference \;antibiotic}$$

### Minimum inhibitory concentration (MIC) of PPE-AgNPs

This technique was performed according to the Clinical and Laboratory Standard Institute 17 guidelines. Shortly, Two-fold serial dilutions of 1/2, 1/4, 1/8, 1/16, 1/32, and 1/64, for the PPE-AgNPs with final concentrations of {1000 (control), 500, 250, 125, 75, 50, and 25} µg/mL were prepared and inoculated into MHA agar welled plates then incubated at 37 °C and 28 °C for 24 h and 72 h for bacteria and fungi, respectively. The minimum inhibitory concentration (MIC) was defined as the lowest concentration of PPE-AgNPs that inhibits bacterial growth^21^.

### Minimum lethal concentration (MLC) of PPE-AgNPs

Based on the results of MIC, Minimum lethal concentration (MLC) is expressed as the values of minimum bactericidal concentration (MBC), and minimum fungicidal concentration (MFC) is presented as the lowest AgNP concentrations inhibiting the growth of pathogenic bacterial and fungal strains. MBC value was performed by sub-culturing all negative MIC wells, then incubating at 37 °C and 28 °C for 24 h and 72 h, respectively. The least concentration that showed no growth on the growth medium was indicated as the MBC value^25^.

### PPE-AgNPs mode of action

Based on MIC, MBC and MFC results. MBC/MIC and MFC /MIC ratios were calculated. PPE-AgNPs had a bactericidal and fungicidal effect when the ratio's value is greater than or equal to 4. On the other hand, it is considered a bacteriostatic or fungistatic agent when the value equals 2 or less^26^.

### SEM preparation for the antimicrobial effect detection of PPE-AgNPs

Sterile distilled water (as control) was prepared in 50 mM PBS (pH 7.0). Subsequently, 100 µL of bacteria or fungi growth preparation (1 × 10^6^ spores or bacteria/mL) were added to each tube and incubated at 150 rpm at 30 and 28 °C for 12 and 24 h for bacteria and fungi, respectively. Then, fungi spores and bacteria were collected thrice after centrifugation (10,000 rpm, 5 min), 4 °C, and washed with 50 mM PBS (pH 7.0). Then, samples were fixed with 3% glutaraldehyde 50 mM PBS (pH 7.0) at room temperature for 4 h without agitation and rinsed with PBS four times for 20 min each. After post-fixing in 1% osmic acid at room temperature for 2 h, the samples were washed with double distilled water for 15 min. Samples were dehydrated through a graded alcohol series of 30%, 50%, 70%, 80%, 90%, and 95%, then thrice at 100% for 15 min in each series. Following this, samples were incubated in isoamyl acetate overnight. Samples were then subjected to a critical point of dry carbon dioxide and coated with gold. Observation using a scanning electron microscope (S-3400N, SEM system, Hitachi, Tokyo, Japan)^27^.

### Gas Chromatography (GC–MS) analysis for PPE

The pomegranate peel (PPE) powder was analyzed using Gas-Chromatography–Mass spectrometry (GC–MS, QQQ 7890B). 10 μL of PPE ethanolic extract were diluted with hexane (≥ 99%, Sigma–Aldrich, Darmstadt, Germany) and injection volume at 2 μL was subjected to column chromatography (Agilent 19091S-433: 1 HP-5MS, 30 m × 250 μm × 0.25 μm). The separative chromatography included QQQ Collision Cell EPC (He Quench Gas with a flow rate of 2.25 mL/min, N_2_ Collision Gas with a flow rate of 1.5 mL/min) was applied. After the sample was subjected, the oven temperature was set at 40–280 °C with a flow rate of 4 °C/min and a post-run of 2 min. Qualitative volatile PPE compounds were compared with their mass involved in library data^28^.

### Statistical analysis

All samples and collected data were statistically analyzed and expressed as means using IBM® SPSS® Statistics software (2017). Tukey’s test at a *P*-value of 0.05 was applied^29^. Each experiment was performed in triplicate, and the results are expressed as mean ± SD.

## Results and discussion

### Biosynthesis and characterization of PPE-AgNPs

**Figure 1:**
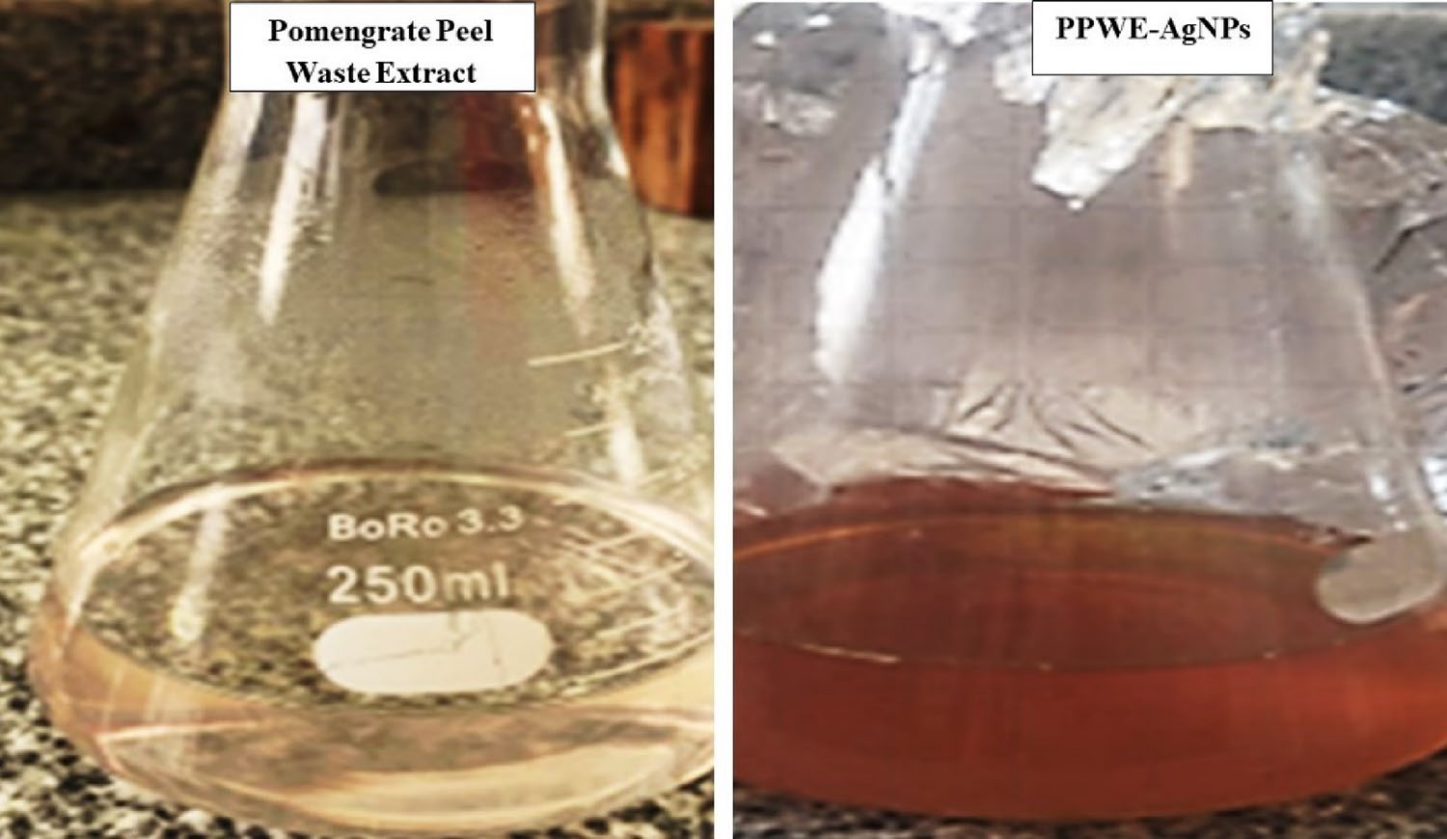
Colour change of PPE solution from *Punica granatum* during reduction of silver nitrate to form PPE-AgNPs. Left: pomegranate peel waste extract. Right: biosynthesis of PPE-AgNPs revealing change in colour from yellow to brown.

PPE-AgNP biosynthesis was detected immediately when the color changed from pale yellow to dark orange upon adding silver nitrate to the pomegranate waste extract under vigorous stirring conditions (Fig. 1). Similar color change observations have been reported in recent studies on green synthesis of silver nanoparticles using pomegranate peel extracts. In a similar study^30^, a color change was noticed from light yellow to brown during biosynthesis of silver nanoparticles from *P. granatum* peel extract after mixing with silver nitrate solution for 20 min. A study reported a color change from pale yellow to dark brown within 1 h of mixing pomegranate peel extract and silver nitrate, indicating silver ion reduction^5^. Similarly, comparable observations were noticed of a color change from light yellow to reddish brown in 5 min after adding silver nitrate to pomegranate peel extract^21,31^. The rapid color change from light to dark shades is attributed to the excitation of surface plasmon vibrations in the synthesized silver nanoparticles. These similar findings across recent literature validate the use of visual color change as a preliminary indicator of successful biosynthesis of silver nanoparticles using pomegranate peel extract.

#### UV–visible spectrophotometer

**Figure 2:**
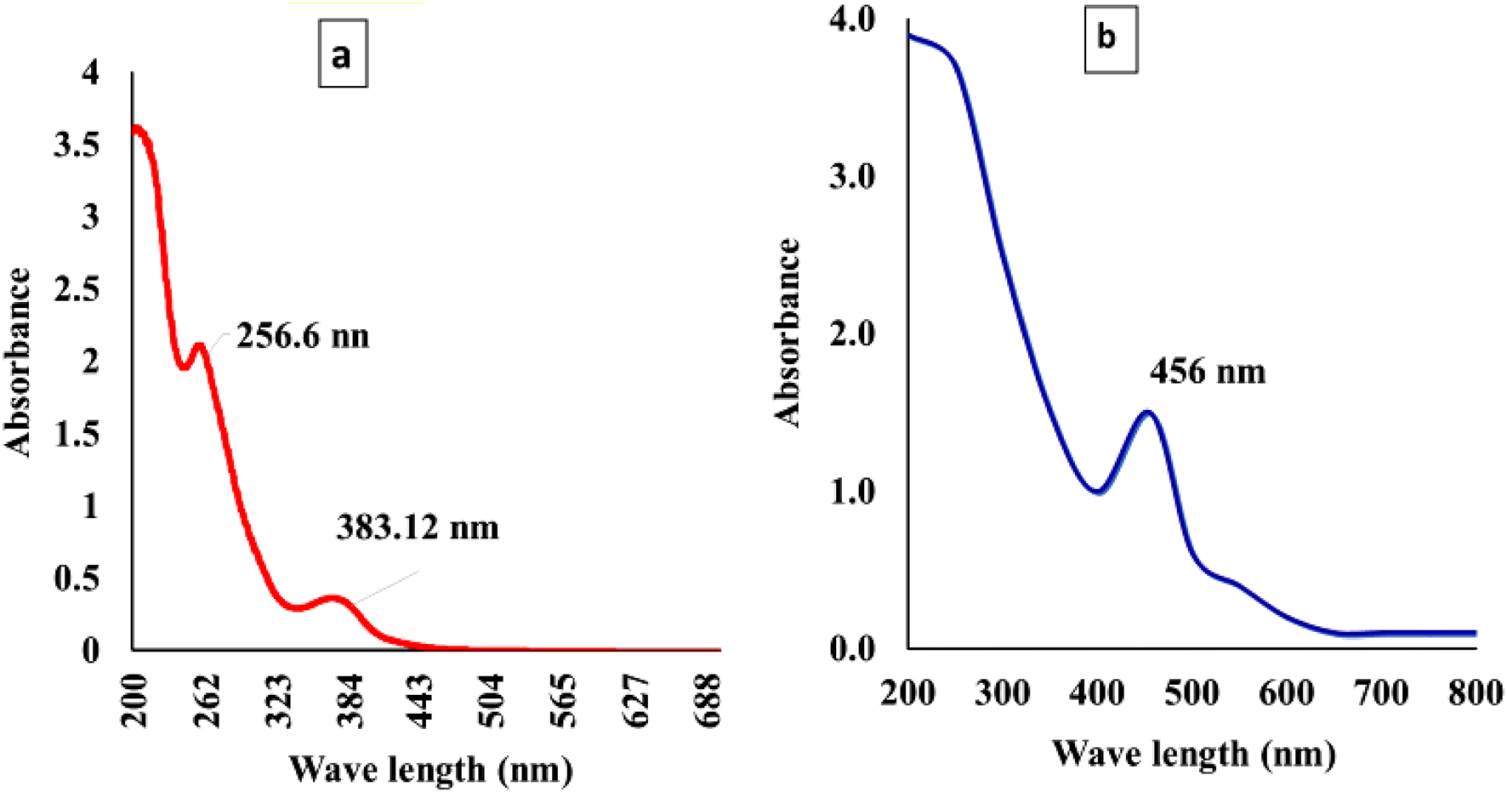
UV–Vis spectrum of synthesized AgNPs from *p.granatum*. (**A**) control treatment, (**B**) AgNPs from mushroom extract.

UV spectrum range were recorded for control PPE and PPE-AgNPs at 200–700 nm. Three spectrum peaks were detected for PPE control at 256.6, and 383.12 nm, while for, PPE-AgNPs, only one peak was detected with a strong surface plasmon resonance (SPR) at 440, recording a score of 0.9 (Fig. 2). Many researchers revealed the detection of AgNPs at 432–442 nm after 5 min of mixing. On the other hand, some studies reported the detection of AgNPs within 10–3 min using different peel extracts^19,32^.

#### DLS and Zeta potential

**Figure 3:**
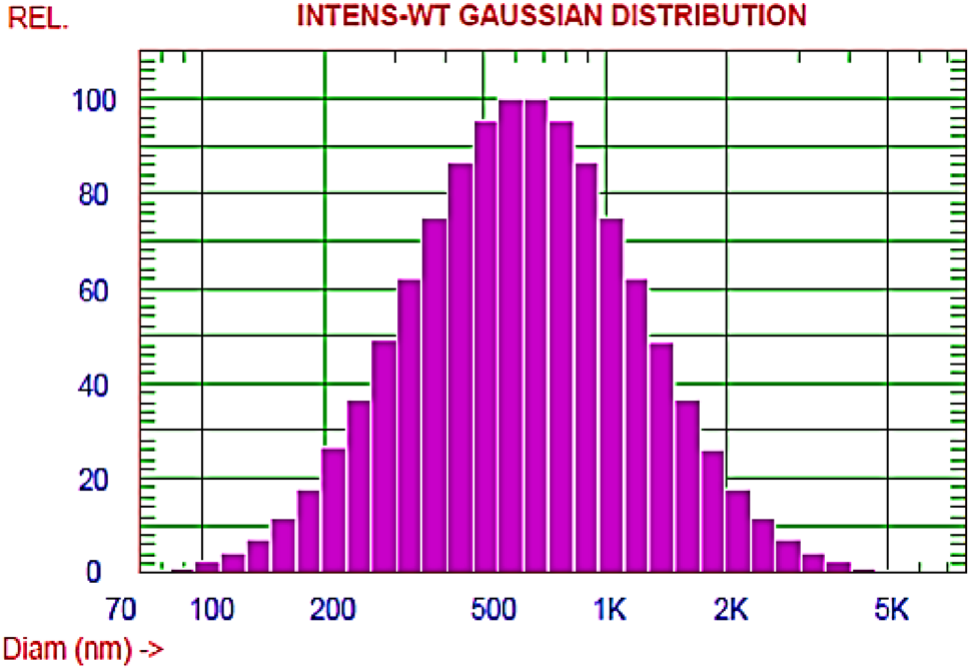
Dynamic light scattering (DLS) analysis for biosynthesized AgNPs from *P. granatum.*

**Figure 4:**
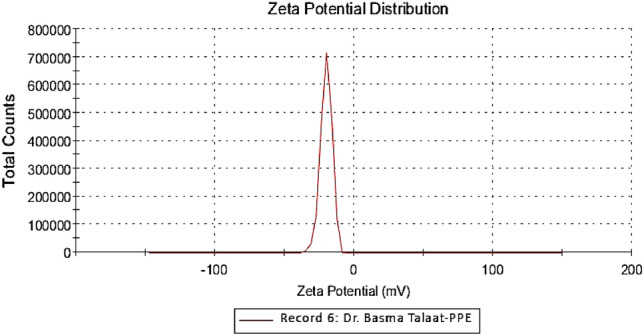
Zeta potential record for PPE-AgNPs from *P. granatum*.

PPE-AgNPs had a diameter ranging from 75.5 to 4085.6 nm with a mean size of 624.4 nm, as shown in Fig. 3 and a zeta potential of − 20.1 mV, as illustrated in Fig. 4. Contrarily,^30^reported a much higher zeta potential of − 68.93 mV and less average particle size distribution between 57.7 and 42.4 nm for the AgNPs synthesized using pomegranate peel extract. Moreover,^33^ found that the average particle size of AgNPs from onion plant leaf extract was 36 nm, and the zeta potential was − 24.1 mV. This wide variation in AgNP sizes is attributed to the reducing agent's structure and the chemical composition of plant leaf extract compounds^34^. The zeta potential value indicated moderate stability of biosynthesized PPE-AgNPs 0.3 mv, which could be attributed to the natural bioactive compounds responsible for capping, reduction of silver ions, and stability of nanoparticles^35^.

#### Fourier transform infrared (FTIR)

**Figure 5:**
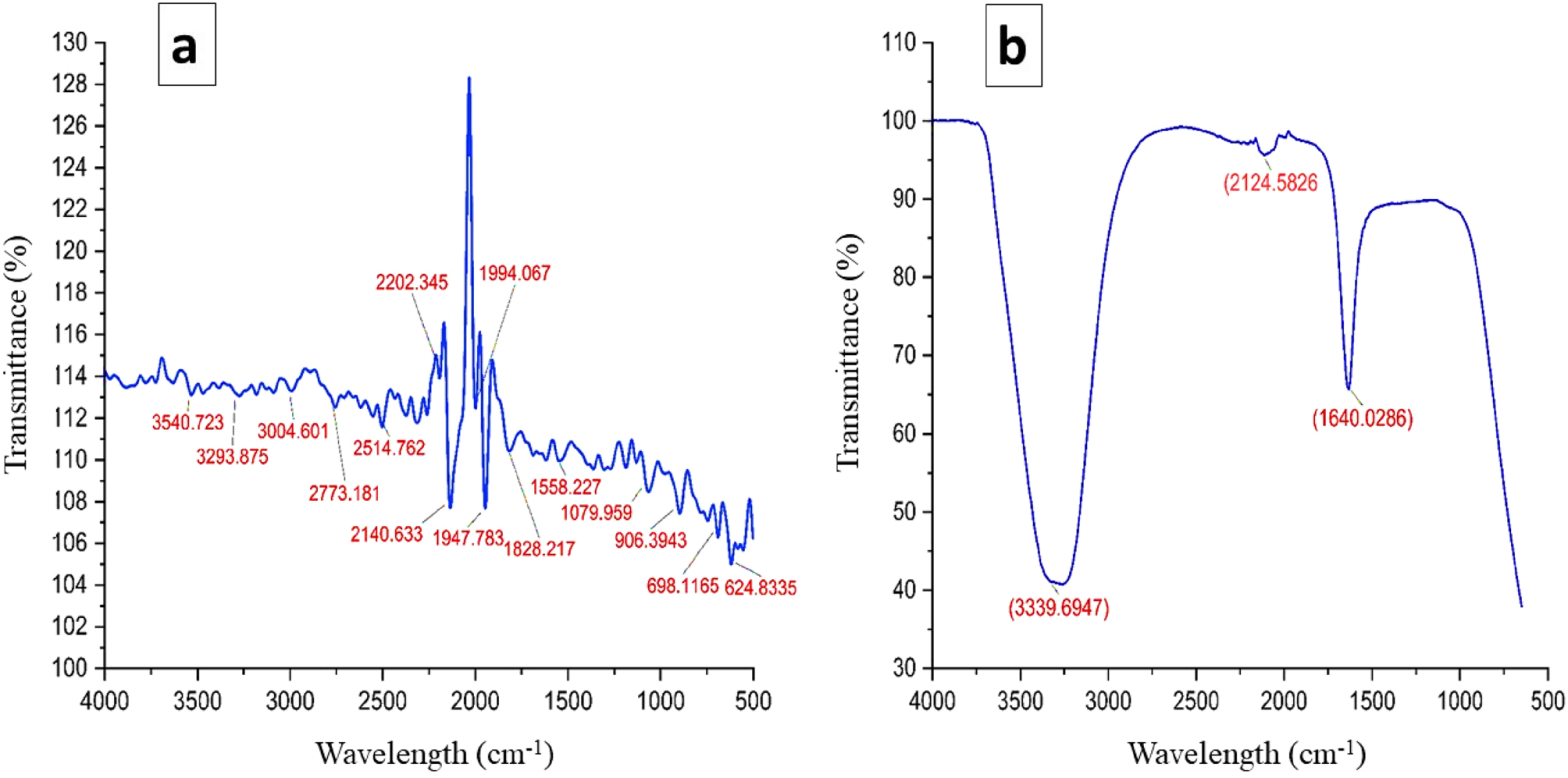
FTIR spectrum of biosynthesized PPE-AgNPs from *P. granatum* by reduction of Ag+ ions. (**A**) Before AgNPs biosynthesis, (**B**) after the AgNPsbiosynthesis.

FTIR was carried out to identify the main biomolecules in plant extracts that help in Ag+ ions reduction to AgNPs. As shown in Fig. 5 and Table 1, the main phytochemicals compounds detected in pomegranate peel waste extract were flavonoids, alkaloids, glycosides, and terpenoids. A similar study by^20^ detected phenolic compounds in pomegranate peel waste extract using FTIR with peaks at 3371/cm, 1635/cm, 1373/cm, and 2924/cm, which were assigned to the stretching of primary and secondary amines, the C–N stretching vibrations of aromatic groups 17, respectively which play a role as capping agents.

**Table 1:** FTIR analysis of biosynthesized PPE-AgNPs from *P.granatum*.

No.	Intensity	Peak position	Functional group	Compound
1	3272.25	3333–3267	C–H stretch	Alkyne
2	2502.81	3300–2500	O–H stretch	Carboxylic acid
3	2192.50	2260–2190	C≡C stretch	Alkyne
4	2135.48	2145–2120	N=C=N stretch	Carbodiimide
5	1999.17	2140–1990	N=C=S stretch	Isothiocyanate
6	1946.18	2000–1900	C=C=C stretch	Alkene
7	1816.53	1815–1770	C= O stretch	Acid chlorides
8	1618.32	1620–1610	C=C stretch	α, β-unsaturated ketone
9	1545.90	1570–1490	NO2 Stretch	Nitro compounds
10	1303.72	1400–1000	C–F Stretch	Alkyl & Aryl halides
11	1186.92	1400–1000	C–F stretch	Alkyl & Aryl halides
12	1065.15	1400–1000	C–F stretch	Alkyl & Aryl halides
13	895.63	900–680	C–H bend	Aromatic compounds
14	689.20	900–680	C–H bend	Aromatic compounds
15	619.20	840–600	C–Cl bend	Alkyl & Aryl halides
16	500.14	600–500	C–I stretch	Aryl halides

Recent studies have comprehensively characterized the diverse phytochemicals present in pomegranate peel waste extracts that contribute to the green synthesis of metallic nanoparticles. A study^36^ detected flavonoids, alkaloids, terpenoids and polyphenols using phytochemical tests and proposed these biomolecules reduce silver ions and stabilize nanoparticles. Specific phenolic acids like gallic acid and ellagic acid using HPLC and hypothesized these phytochemicals provide dual functions of reduction and capping in biosynthesis of gold nanoparticles^36^. Detected peaks at 3396, 3278, and 3169/cm that were attributed to various (OH) and (NH) groups as well as H-bonding water–OH, alcohols, and amides that possibly are a part of NPs-stabilizing compounds, and the capping agents. Hence, it is reported that the amide group, amino, carbonyl group, and polyphenolic compounds in the PPE are a part of the redox reaction, dispersion, capping, and stabilizers involved in the production of nanoparticles during the process of synthesis^37^. In summary, these recent studies strongly indicate the diverse phytochemical composition of pomegranate peel that provides reducing, capping and stabilizing agents for green sustainable of nanoparticle synthesis.

#### Scanning electron microscope (SEM)

**Figure 6:**
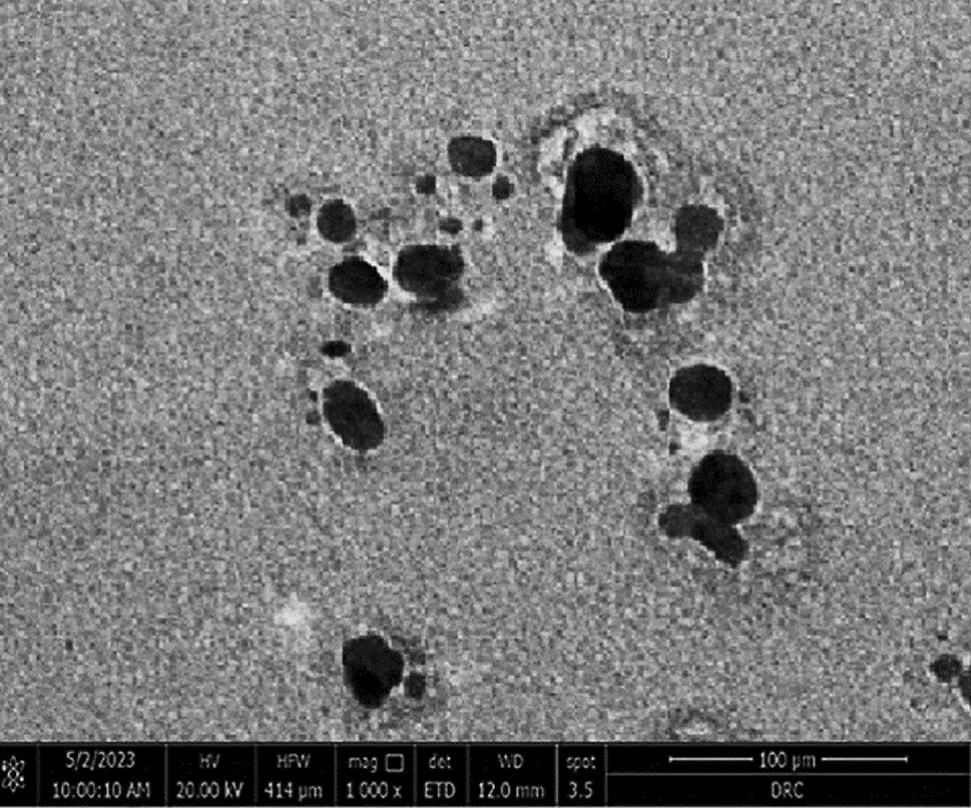
Scanning Electron Microscope (SEM) indicating the biosynthesized PPE-AgNPs from *P. granatum.*

Scanning Electron Microscopy (SEM) determined the PPE-AgNP’s shape and size from *Punica granatum*. SEM analysis showed semi-spherical coated AgNPs, as shown in Fig. 6.

### Inhibitory activity of PPE-AgNPs from *Punica granatum*

Antibiotic susceptibility test of the biosynthesized PPE-AgNPs was performed using eight foodborne pathogens, including six bacterial and two fungal strains. Data in Table 2 showed that all tested pathogenic bacteria were highly sensitive to AgNPs with IZD ranging from 4.5 to 0.96 cm, while fungi were resistant with the lowest IZD of 0.96 cm when compared to the control antibiotic drug with IZD scored 4.1–0.96 cm. The most sensitive bacterial strain was recorded for *B. subtilis* with an IZD of 4.5 cm and activity index (AI) of 1.10. However, the lowest IZD and activity index (AI) was observed for the fungal strains of *A. flavus and R. oryzae* ATCC 96382, reaching 1.30 and 0.96 with AI of 0.48 and 0.51, respectively. This inhibitory activity is attributed to the binding of silver to the phospholipid layer of the bacterial cell membrane, where the formation of pores occurs. This, in turn, results in their destabilization and an increase in membrane permeability, causing leakage of cellular metabolites and cell death^30,38^. Also,^34^ found that the AgNPs synthesized using pomegranate fruit peel extract as a reducing agent exhibited a fairly significant inhibition with 2.6 cm against *S. aureus.* The major mechanism of silver nanoparticles manifesting antibacterial properties was anchoring or penetrating the bacterial cell wall and modulating cellular signalling by dephosphorylating peptide substrate on tyrosine residues.

**Table 2:** Inhibitory activity PPE-AgNPs expressed as zone diameter (IZD) and activity index (AI).

Pathogen strains	Inhibition zone diameter (cm)		AI
	Antibiotic (1000 µg/mL)	PPE-AgNPs (1000 µg/mL)	
G^+ve^ Bacteria			
*B. subtilis* ATCC 6633	4.10^b^ ± 0.41	4.5^a^ ± 0.41	1.10
*E. faecalis* ATCC 29212	0.96^g^ ± 0.22	2.5^d^ ± 0.55	2.60
G^−ve^ Bacteria			
*E. coli* ATCC 8379	0.98^g^ ± 0.16	2.0^e^ ± 0.14	2.05
*K. pneumoniae* ATCC 00607	1.30^g^ ± 0.65	2.5^d^ ± 0.77	1.92
*S. typhi* DSM 17058	0.96^h^ ± 0.02	2.6^d^ ± 0.11	2.70
*S. sonnei* DSM 5570	0.98^h^ ± 0.04	2.5^d^ ± 0.30	2.55
Fungi			
*A. flavus* ATCC 9643	2.77^c^ ± 0.02	1.30^g^ ± 0.84	0.48
*R. oryzae* ATCC 96382	1.88^ef^ ± 0.50	0.96^h^ ± 0.45	0.51

Standard antibiotics were streptomycin, ampicillin, and fluconazole against G^+ve^ bacteria, G^−ve^ bacteria and fungi, respectively.*mm, milli meter; AI, activity index; and SE ( ±), standard error. Values in the same column followed by the same letter are not significantly different, according to^29^ at a 5% level.

### Minimum inhibition concentration (MIC) of PPE-AgNPs

MIC values of PPE-AgNPs against the tested pathogenic bacterial and fungal strains ranged from 1000 to 12.5 μg/mL, as illustrated in Table 3. The MIC value was exhibited at 250 μg/mL for* E. coli* ATCC 8379 and *E. faecalis ATCC 29212*, while it was 125 μg/mL* K. pneumoniae*. On the other hand, it was 50 μg/mL against *S. typhi* and 25 μg/mL against* B. subtilis*. The results showed 100% of the antibacterial spectrum activity of PPE-AgNPs at concentrations ranging from 1000 to 250 μg/mL, whereas at concentrations of 125, and 75 μg/mL the activity was 66.7% and 50%, respectively. Whereas at a concentration of 50 μg/mL, the antibacterial spectrum showed 33.3%; at a 25 μg/mL concentration, it exhibited 16.6% activity. There was no antibacterial spectrum at 12.5 μg/mL. The PPE-AgNPs MIC antifungal activity investigation was at concentrations of 1000–12.5 μg/mL, both *A. flavus* and *R. oryzae*. The MIC was 250 μg/mL for *R. oryzae* and 75 μg/mL for *A. flavus*. At 1000–250 μg/mL concentrations, 50% of the spectrum of activity was attained for all fungi at 125–75 μg/mL. In addition, concentrations ranging from 25 to 12.5 μg/mL did not display any activity against the tested fungal strains.

**Table 3:** Minimum inhibitory concentration (MIC) of PPE-AgNPs against foodborne pathogens.

Pathogenic bacteria	MIC (µg/mL) of PPE-AgNPs							
	1000 (control)	500	250	125	75	50	25	12.5
*B. subtilis* ATCC 6633	−	−	−	−	−	−	−	+
*E. faecalis* ATCC 29212	−	−	−	+	+	+	+	+
*E. coli* ATCC 8379	−	−	−	+	+	+	+	+
*K. pneumoniae* ATCC 00607	−	−	−	−	+	+	+	+
*S. typhi* DSM 17058	−	−	−	−	−	−	+	+
*S. sonnei* DSM 5570	−	−	−	−	−	+	+	+
Spectrum of activity (%)	6/6	6/6	6/6	4/6	3/6	2/6	1/6	0/6
	100	100	100	66.7	50.0	33.3	16.6	0

Pathogenic fungi	MIC (µg/mL) of PPE-AgNPs							
	1000 (control)	500	250	125	75	50	25	12.5
*A. flavus* ATCC 9643	−	−	−	−	−	+	+	+
*R. oryzae* ATCC 96,382	−	−	−	+	+	+	+	+
Spectrum of activity (%)	2/2	2/2	2/2	1/2	1/2	0/2	0/2	0/2
	100	100	100	50	50	0.00	0.00	0.00

−,  no growth; +, growth.

### Minimum lethal concentration (MLC) of PPE-AgNPs

The minimum lethal concentration MLC (MBC and MFC) for pomegranate peel silver nanoparticles (PPE-AgNPs) are presented in Table 4. The MBC value was 500 μg/mL for* E. coli*, *E. faecalis* and *K. pneumoniae*. On the other hand, it was 250 μg/mL for *S. sonnei* and 125 μg/mL for *S. typhi* and *B. subtilis*. The results showed 100% of the antibacterial spectrum activity of PPE-AgNPs at concentrations ranging from 1000 to 250 μg/mL, whereas at concentrations of 250–125 μg/mL, the activity was 33.3%. There was no antibacterial spectrum at a 75–12.5 μg/mL concentration. The MFC was 1000 μg/mL for *R. oryzae* ATCC 96382, while it was 500 μg/mL for *A. flavus*, as shown in Table 4. At the concentration 1000, the spectrum activity was 100%, while at 500 μg/mL, only 50% of the activity was attained for all fungi at 250–12.5 μg/mL did not display any activity against the tested fungal strains.

**Table 4:** Minimum lethal concentration (MLC) of PPE-AgNPs against foodborne pathogens.

Pathogenic bacteria	MBC (µg/mL) of PPE-AgNPs							
	1000 (control)	500	250	125	75	50	25	12.5
*B. subtilis* ATCC 6633	−	−	−	−	+	+	+	+
*E. faecalis* ATCC 29212	−	−	+	+	+	+	+	+
*E. coli* ATCC 8379	−	−	+	+	+	+	+	+
*K. pneumoniae* ATCC 00607	−	−	+	+	+	+	+	+
*S. typhi* DSM 17058	−	−	−	−	+	+	+	+
*S. sonnei* DSM 5570	−	−	−	+	+	+	+	+
Spectrum of activity (%)	6/6	6/6	2/6	2/6	0/6	0/6	0/6	0/6
	100	100	33.3	33.3	0	0	0	0

Pathogenic fungi	MFC (µg/mL) of PPE-AgNPs							
	1000 (control)	500	250	125	75	50	25	12.5
*A. flavus* ATCC 9643	−	−	+	+	+	+	+	+
*R. oryzae* ATCC 96382	−	+	+	+	+	+	+	+
Spectrum activity (%)	2/2	1/2	0/2	0/2	0/2	0/2	0/2	0/2
	100	50	0.00	0.00	0.00	0.00	0.00	0.00

−,  no growth, +, growth.

### Pomegranate peels silver nanoparticles (PPE-AgNPs) action mode

**Figure 7:**
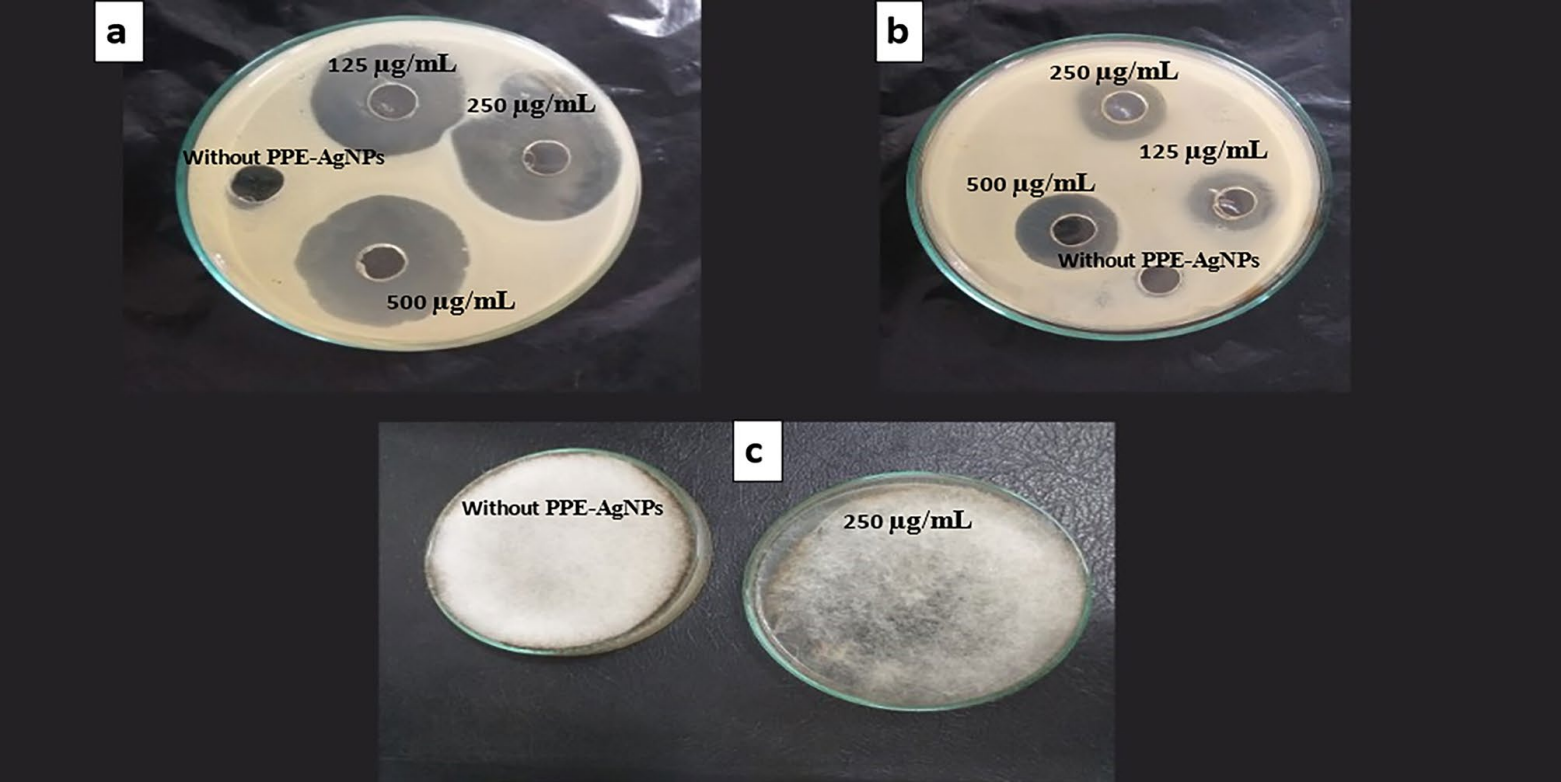
Antimicrobial activity of PPE-AgNPs against the most sensitive pathogenic strains, (**a**) *B. subtilis,* (**b**) *K. pneumonia*, (**c**) *R. oryzae.*

Finally, it could be observed that the mode of action of PPE-silver nanoparticles against pathogenic bacterial and fungal strains is shown in Table 5. Results indicated that the PPE-AgNPs have a bactericidal against *E. coli* and *E. faecalis*, while it was bacteriostatic with* B. subtilis*,* K. pneumoniae*, *S. typhi*, *S. sonnei*, and *S. sonnei*. Also, it revealed a fungistatic effect for both fungal strains. In Brief, PPE-AgNPs showed high antimicrobial activity against all tested strains. The highest inhibition activity of PPE-AgNPs was recorded for the *B. subtilis* strain followed by *K. pneumonia*, while the highest resistance was noticed for *R. oryzae,* Fig. 7*.* Previous research has elucidated the complex interactions between nanoparticles (NPs) and microorganisms including bacteria, fungi and viruses along with the resultant antimicrobial effects. Metal NPs like silver, gold, zinc oxide and copper have shown pronounced antibacterial activity against both Gram positive and Gram negative bacteria due to their positively charged surfaces interacting and damaging the negatively charged bacterial cell membrane leading to cell death^39,40^. The antimicrobial mechanisms involve generation of reactive oxygen species, inhibition of vital microbial enzymatic and protein functions, and disruption of DNA replication and protein synthesis causing growth inhibition. Factors like NP size, shape, surface properties and concentration influence the antibacterial effects, with smaller NPs having greater impacts due to higher surface area to volume ratios^41^. However, some studies have reported microbial resistance development against NPs indicating dynamic interactions and adaptation. Overall, previous research has clearly demonstrated the broad-spectrum antimicrobial properties of diverse NPs and elucidated their complex interactions with microbial cells that induce cell damage and growth inhibition. But microbial adaptation mechanisms point to the need for further studies on these evolving NP-microbe interactions***.***

**Table 5:** Minimum inhibitory concentration (MIC) and minimum lethal concentration (MLC) of PPE-AgNPs against foodborne pathogens.

Pathogenic bacteria	MIC (PPE-AgNPs µg/mL)	MLC (PPE-AgNPs µg/mL)	MLC/MIC ratio	Mode of action
Bacteria				
* E. faecalis* ATCC 29212	250	500	2.0	+
* E. coli* ATCC 8379	250	500	2.0	+
* B. subtilis* ATCC 6633	25	125	5.0	−
* K. pneumoniae* ATCC 00607	125	500	4.0	−
* S. typhi* DSM 17058	50	125	2.5	−
* S. sonnei* DSM 5570	75	250	3.3	−
Fungi				
* A. flavus* ATCC 9643	75	500	6.6	−
* R. oryzae* ATCC 96382	250	1000	4.0	−
Bactericidal/Fungicidal (+) = ≤ 2 and Bacteriostatic/fungistatic (−) effect = ≥ 4

### SEM preparation for the antimicrobial effect detection of PPE-AgNPs

**Figure 8:**
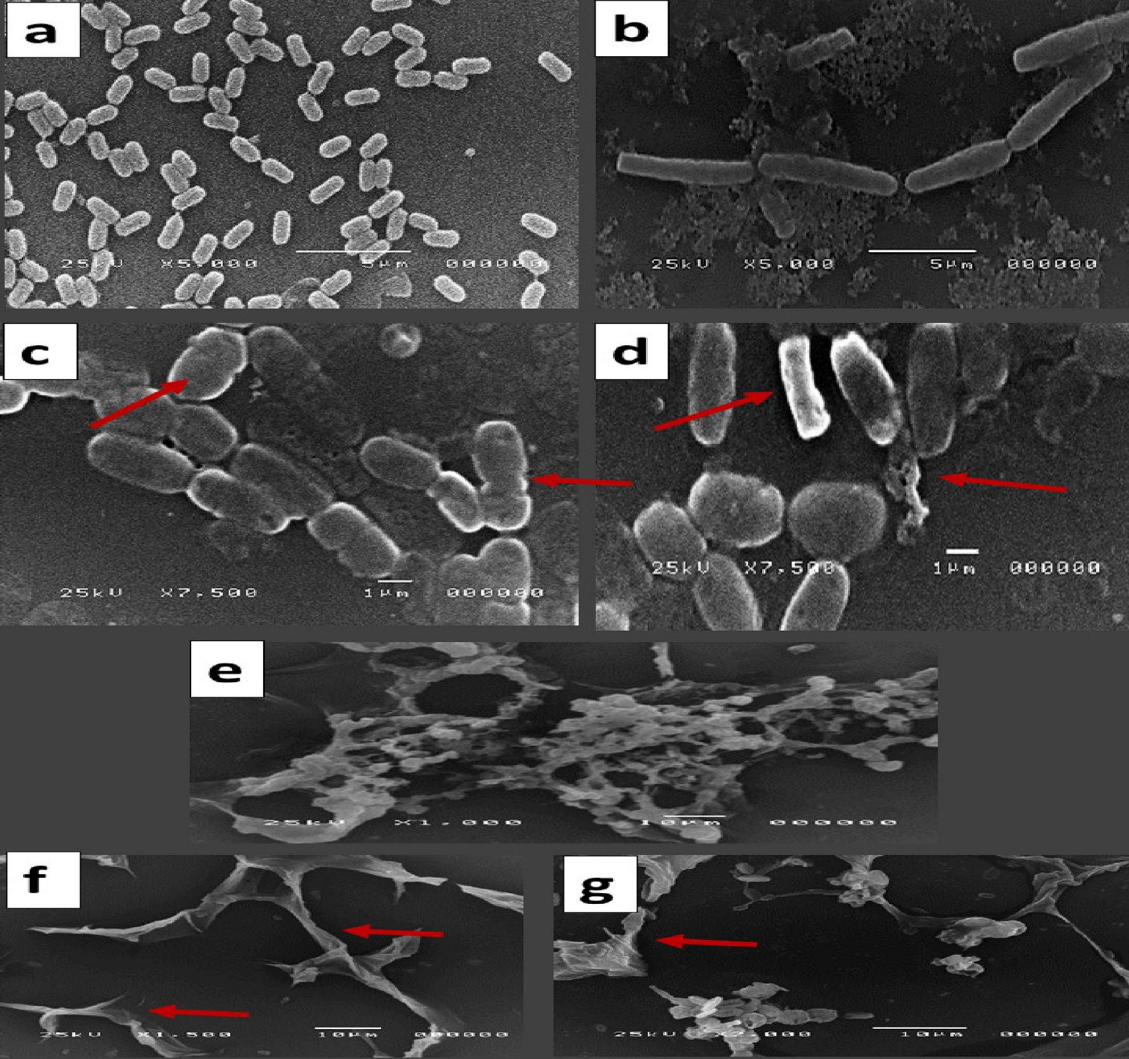
SEM images of (**a**) *Bacillus subtilis*, (**b**) *Enterococcus faecalis*, and (**e**) *Aspergillus niger* cells control treated with distilled water, while (**c**) *Bacillus subtilis*, (**d**) *Enterococcus faecalis*, and (**e, f**) *Aspergillus niger* cells treated with 1000 µg/mL PPE-AgNPs. Arrows refer to the morphologic alterations in cells and contact of PPE-AgNPs cell membrane and collapsed cell walls.

In our SEM findings, obvious signs of differences in cell morphology between control and treated cells with damage to the cell wall for the most influenced microorganisms of *Bacillus subtilis*, *Enterococcus faecalis*, and *Aspergillus niger* were found according to analysis with the aid of a scanning electron microscope (SEM). After 12 and 24 h of incubation at 30 and 28 °C for 12 and 24 h for bacteria and fungi, respectively. The control microbes treated with distilled water displayed a normal and compact morphology (Fig. 8a, b, e), whereas the microbial cells treated with 1000 µg/mL PPE-AgNPs showed irregular modifications. As shown in Fig. 8c, d, cell surfaces appeared to suffer from PPE-AgNPs with irregular morphological shapes, disrupted cell walls and pores or invaginations on cells, and hypha broken down for fungi, as shown in Fig. 8f, g. In the same line, it was found that AgNPs, there was accumulation on *S. aureus* and *P. aeruginosa* cell surface, which results in pores or invaginations on cells that affect bacterial viability^27,42^. Cells treated with AgNPs showed destruction and distortion at cell poles due to an accumulation of nanoparticles that affected cell viability and caused cell death and pores or invaginations on cells. The antifungal SEM investigation^43^ shows a great variation between a control with the normal shape of fungal mycelium, whereas the treated sample with AgNPs shows aggregations of small micro nanoparticles at an earlier stage of formation on the hyphae^44^.

### Gas Chromatography (GC–MS) analysis for PPE

**Figure 9:**
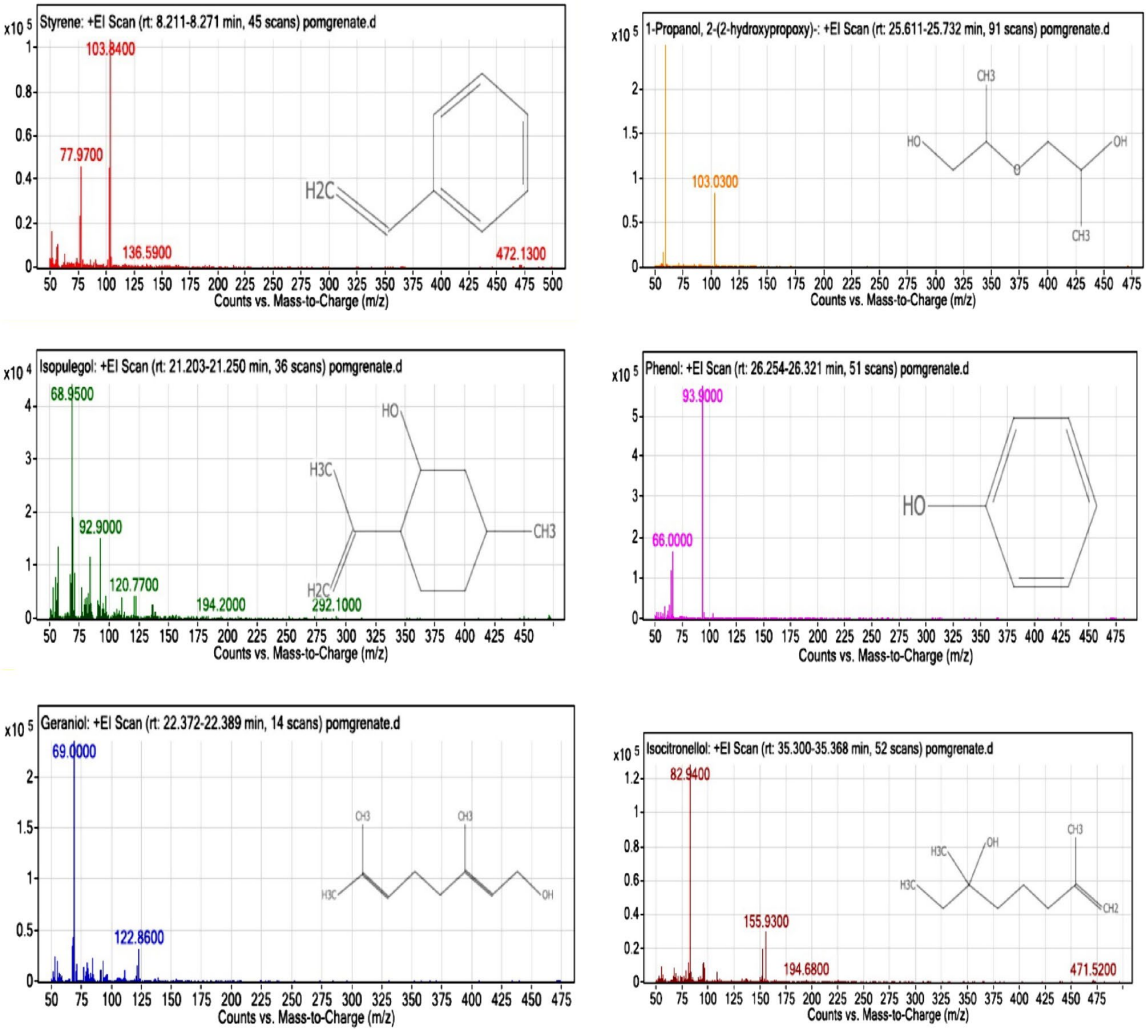
Chromatographic analysis of bioactive compounds in pomengrate peel (PPE).

The phytochemical composition of pomegranate peel (PPE) was analyzed using GC–MS and presented in Table 6 and Fig. 9. The major constituents of pomegranate peel were phenol (51.1%), followed by Isocitronellol (19.41%) and 1-Propanol, 2-(2-hydroxypropyl)- (16.05%). Additionally, other terpenoids such as geraniol (4.89%), styrene (4.47%), and isopolygol (4.07%) have been identified in the ethanolic extract of pomegranate peel (Fig. 9). Geraniol is a monoterpenic alcohol with a pleasant rose-like aroma, is found in many essential oils and has antibacterial and antifungal potential^45,46^. Phenolic compounds have gained attention, especially in the food research sector, as microbial growth inhibitors for foodborne pathogenic and spoilage bacteria. All phenols also have promising anti-quorum sensing potential and inhibit the biofilm formation and toxin production of food-related pathogens^47^.

**Table 6:** Chemical composition (%) of PPE analyzed by GC–MS.

No. peak	Component names	RT^a^ (min)	Percentage (%)^b^
1	Styrene	8.241	4.47
2	Isopolygol	21.242	4.07
3	Geraniol	22.376	4.89
4	1-Propanol, 2-(2-hydroxypropoxy)-	25.681	16.05
5	Phenol	26.312	51.1
6	Isocitronellol	35.308	19.41

^a^Retention time.

^b^Compound percentage.

## Conclusion

The present study demonstrates a simple, eco-friendly biosynthesis of PPE-AgNPs using pomegranate peel extract as a reducing agent. The phytochemicals detected by the FTIR, such as flavonoids, alkaloids, glycosides, and terpenoids, acted as better reducing and capping agents. Besides the physicochemical advantages of PPE-AgNPs, these have shown improved antimicrobial activity, which can contribute to solving the global problem of multi-drug resistant (MDR) microorganisms soon.

## Data Availability

The raw and analyzed data used during the current study are available from the corresponding author upon reasonable request. All microbial pathogens were provided by the Agricultural Microbiology Department, Faculty of Agriculture, Ain Shams University, Cairo, Egypt and were deposited in the following strain providers (1) *B. subtilis* ATCC 6633 was from ATCC collection https://www.atcc.org/products/6633. (2) *E. fecalis* ATCC 29,212 was from the ATCC collection https://www.atcc.org/products/29212. (3) *E. coli* ATCC 8379 was from ATCC collection https://www.atcc.org/products/8379 and was deposited in GenBank with taxonomy ID: NCBI:txid 481805 https://www.ncbi.nlm.nih.gov/Taxonomy/Browser/wwwtax.cgiTPE-AgNPs id = 481805. (4) *K. pneumoniae* ATCC 00607 was from ATCC collection https://www.atcc.org/products/00607. (5) *S. typhi* DSM 17058 was from DSM collection https://www.dsmz.de/collection/catalogue/details/culture/DSM-17058. (6) *S. sonii* DSM 5570 was from DSM collection https://www.dsmz.de/collection/catalogue/details/culture/DSM-5570. (7) *A. flavus* ATCC 9643 was from ATCC collection https://www.atcc.org/products/9643. (8) *R. oryzae* ATCC96382 was from ATCC collection https://www.atcc.org/products/96382. (9) Policy of plant materials collection https://portals.iucn.org/library/efiles/documents/PP-003-En.pdf.
